# Platypnea-Orthodeoxia Syndrome in Relation to Severe SARS-CoV-2 Infection

**DOI:** 10.7759/cureus.79682

**Published:** 2025-02-26

**Authors:** Teresa Valido, Ana Carolina Chumbo, Filipa Figueiredo, Teresa Cruz

**Affiliations:** 1 Internal Medicine, Unidade Local de Saúde (ULS) Amadora/Sintra, Amadora, PRT

**Keywords:** covid-19, covid-19 pandemic, platypnea-orthodeoxia syndrome, pulmonary fibrosis, sars-cov-2

## Abstract

Platypnea-orthodeoxia syndrome (POS) is characterized by dyspnea and oxygen desaturation in the upright position, caused by arteriovenous shunts (intracardiac, intrapulmonary, or physiological). In recent years, POS has been described in patients with SARS-CoV-2 infection, in both acute and rehabilitation phases. This case describes a 90-year-old man who presented to the emergency department with a dry cough and dyspnea and was diagnosed with severe SARS-CoV-2 infection. He underwent non-invasive ventilation for refractory hypoxemia. During convalescence, CT scans revealed fibrotic changes predominantly in the lung bases. He experienced persistent dyspnea and oxygen desaturation upon standing. Blood gas analysis confirmed orthodeoxia. After ruling out intracardiac and intrapulmonary shunts, secondary POS due to SARS-CoV-2 infection was presumed. He continued rehabilitation with progressive improvement, resolving the condition a month after discharge. This phenomenon, likely induced by fibrotic sequelae of SARS-CoV-2 in the lung bases causing a physiological shunt, highlights a potentially underdiagnosed complication of interstitial lung diseases.

## Introduction

Platypnea-orthodeoxia syndrome (POS) is a rare clinical entity characterized by positional dyspnea (platypnea) and oxygen (O2) desaturation (orthodeoxia) associated with standing, which resolves in the supine position. It is defined by a decrease of 4 mmHg in arterial oxygen pressure (PaO2) and 5% in peripheral oxygen saturation (SpO2) in orthostatism [1].

The pathophysiology of this syndrome, still not fully understood, seems to be related to the presence of a shunt between arterial and venous blood. The main cause of POS is an intracardiac shunt, particularly in the presence of a patent foramen ovale or another atrial septal defect. POS can also occur in pulmonary shunts, such as intrapulmonary shunts (e.g., arteriovenous malformations, hepatopulmonary syndrome, or acute respiratory distress syndrome (ARDS)), or in conditions affecting ventilation-perfusion (V/Q) matching, such as diseases impacting the lung parenchyma predominantly at the bases (e.g., interstitial diseases, chronic obstructive pulmonary disease (COPD), or pneumonectomy). Additionally, rare cases have been reported due to other causes such as amiodarone-induced pulmonary toxicity, bronchial stenosis caused by radiation, and organophosphate toxicity or in association with other pathologies such as diabetic neuropathy and Parkinson's disease [1]. 

Diagnosing POS requires a high degree of suspicion, given the presentation that may range from subtle to pronounced [2]. After detecting peripheral O2 desaturation greater than 5% in the upright position with improvement in the supine position, identifying the cause is essential. Since intracardiac causes are the most common, an echocardiogram with agitated saline injection is the first recommended test. It should be performed both sitting and supine, as the appearance of bubbles in the left atrium during the first three cardiac cycles indicates an intracardiac shunt, while late appearance in the left chambers suggests an extracardiac shunt, most commonly in the pulmonary vasculature. In the absence of abnormalities on echocardiography, the next step should be a chest CT scan to investigate intrapulmonary changes. If no abnormalities are detected in these tests, less common causes should be considered [2].

This case report describes a patient diagnosed with POS during the rehabilitation phase after severe COVID-19, associated with fibrotic pulmonary changes secondary to SARS-CoV-2 infection.

## Case presentation

**Figure 1:**
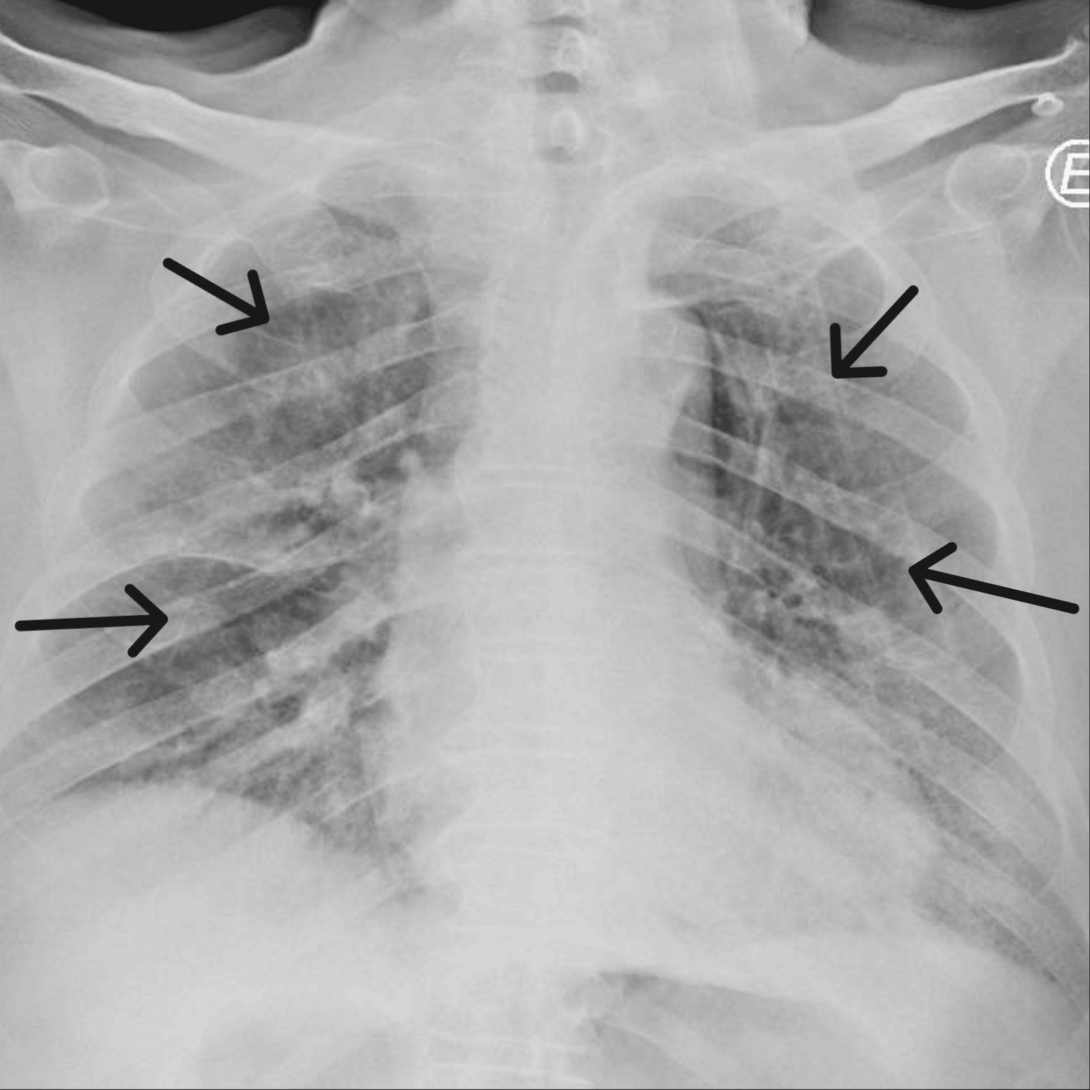
Chest X-ray upon admission showing bilateral infiltrates

A 90-year-old man, independent in daily activities, with a medical history of essential hypertension and treated with an angiotensin-converting enzyme inhibitor and a thiazide diuretic, was admitted to the emergency department with a two-day history of dry cough, dyspnea, and fatigue during minor exertion. Upon admission, he was tachypneic with an SpO2 of 80% in room air. Arterial blood gas analysis on 4 L/min oxygen therapy via nasal cannula revealed type 1 respiratory failure, with a PaO2/FiO2 ratio of 145 and hyperlactatemia (Table 1). Chest X-ray showed bilateral interstitial infiltrates (Figure 1). Testing for SARS-CoV-2 nucleic acids was positive, and the patient was admitted with a diagnosis of severe COVID-19.

**Table 1:** Laboratory tests done at admission and at worsening after 25 days of hospitalization

	Upon admission in the emergency room (FiO2 21%)	Worsening in the ward at day 25 (FiO2 31%)	Reference values
Arterial blood gas analysis			
pH	7.445	7.416	7.350-7.450
Partial pressure of carbon dioxide (PaCO2)	37 mmHg	40 mmHg	35-45 mmHg
Partial pressure of oxygen (PaO2)	46.5 mmHg	75.3 mmHg	70-100 mmHg
Bicarbonate (HCO3)	25 mmol/L	25.1 mmol/L	21-26 mmol/L
Lactate	2.550 mmol/L	1.420 mmol/L	<1.800 mmol/L
Oxygen saturation (SaO2)	82%	94.2%	>96%
PaO2/FiO2 ratio	145	243	>300
Inflammatory markers			
C-reactive protein	7.05 mg/dL	5.65 mg/dL	<0.50 mg/dL
D-dimer	881 ug/L	>35,000 ug/L	<500 ug/L

During his stay in the general ward, the patient's clinical status deteriorated; he developed refractory hypoxemia and was transferred to an intermediate care unit where he started on non-invasive ventilation (NIV). Chest CT revealed interstitial pneumonia affecting 60% of the parenchyma and areas of consolidation with air bronchograms. He received a seven-day course of ceftriaxone 2 g/day and corticosteroid therapy with methylprednisolone 1 mg/kg/day. A follow-up chest CT performed due to difficulty weaning off NIV revealed persistent irregular subpleural reticular opacities with an organizing component. Assuming organizing pneumonia, corticosteroid therapy was intensified. He remained on NIV for 20 days before being transferred back to the general ward on oxygen therapy via a Venturi mask.

**Figure 2:**
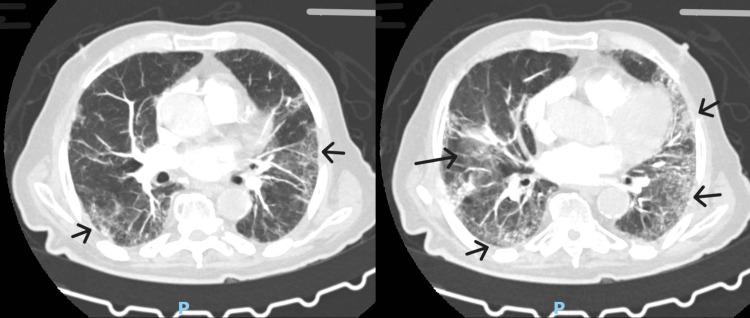
Chest CT done in the ward, showing fibrotic changes predominantly at the lung bases

In the ward, after discontinuation of NIV, but due to subsequent worsening hypoxemia requiring a new increase in oxygen supplementation (Table 1), a contrast-enhanced chest CT identified bilateral segmental pulmonary embolism, and anticoagulation therapy was initiated. The CT also revealed permeability abnormalities in the lung parenchyma, with reticular consolidations predominantly at the bases, consistent with fibrotic changes (Figure 2).

While on anticoagulation and undergoing respiratory rehabilitation, the patient exhibited intolerance to sitting upright even with supplemental oxygen, reporting severe dyspnea and significant oxygen desaturation when seated (SpO2 92-94% in the supine position, dropping to 65-75% when seated). Blood gas analysis in both supine and seated positions confirmed orthodeoxia. A transthoracic echocardiogram with agitated saline injection showed no atrial shunt, and a reassessment of thoracic CT images found no arteriovenous malformations.

The diagnosis of POS secondary to SARS-CoV-2-induced pulmonary permeability changes was established. The patient continued respiratory physiotherapy during hospitalization, with gradual improvement. At discharge after 69 days of hospitalization, he still experienced platypnea and orthodeoxia, warranting continued home rehabilitation. Oxygen therapy was eventually discontinued three months after admission, with satisfactory tolerance in the upright position.

## Discussion

This article presents a clinical case of POS secondary to fibrotic changes caused by SARS-CoV-2 pneumonia. There's already evidence suggesting the existence of many long-term sequelae in survivors of SARS-CoV-2 infection [3,4]. Pulmonary fibrotic changes have been detected three weeks after symptom onset, regardless of disease severity [3]. Alterations in pulmonary function, including obstructive, restrictive, or mixed patterns, have also been described. These sequelae are more common in patients with severe COVID-19 and significant elevation of inflammatory markers during the acute phase [3].

Literature indicates that COVID-19, in both its acute phase and post-recovery, can present with POS [5,6]. Most cases have been reported in patients with ground-glass opacities predominantly at the lung bases, causing ventilation deficiencies that exacerbate in the upright position. This is further aggravated by microangiopathy in the pulmonary vasculature, a frequent complication of COVID-19 [7].

This syndrome is likely underdiagnosed in the context of post-COVID-19 sequelae due to its subtle presentation, prolonged bed immobilization, and the comorbidities commonly associated with the disease, such as pulmonary embolism, which also presents with difficulties in weaning off oxygen therapy. Rehabilitation plays a crucial role in improving or resolving this syndrome [8], both respiratory rehabilitation and physical physiotherapy, and the potential therapeutic role of antifibrotic drugs in these cases warrants further study. It is important to highlight that POS is a significant contributor to morbidity and prolonged hospitalizations in patients with post-COVID-19 sequelae [9] and its diagnosis should be considered in all patients with upright intolerance and prolonged oxygen therapy dependency.

## Conclusions

The presented case demonstrates that SARS-CoV-2-induced pulmonary changes, including fibrotic alterations and ventilation-perfusion mismatch, can lead to the development of POS, significantly impacting recovery and quality of life. Timely diagnosis and appropriate management, including respiratory rehabilitation, were pivotal in improving the patient's condition, although residual symptoms persisted. The findings emphasize the need for heightened awareness of POS in post-COVID-19 patients, particularly those with severe disease, prolonged immobilization, or unexplained oxygen therapy dependency. 
